# Development and Initial Evaluation of a Nurse-Led Healthcare Clinic for Homeless and At-Risk Populations in Tasmania, Australia: A Collaborative Initiative

**DOI:** 10.3390/ijerph182312770

**Published:** 2021-12-03

**Authors:** Grace Bennett-Daly, Maria Unwin, Ha Dinh, Michele Dowlman, Leigh Harkness, Jane Laidlaw, Kathleen Tori

**Affiliations:** 1 School of Nursing, University of Tasmania, Newnham, Launceston, TAS 7248, Australia; maria.unwin@utas.edu.au (M.U.); thithuyha.dinh@utas.edu.au (H.D.); michele.dowlman@utas.edu.au (M.D.); leigh.harkness@utas.edu.au (L.H.); Kathleen.Tori@utas.edu.au (K.T.); 2 Mission Health Nurse-Led Clinic, Launceston, TAS 7250, Australia; jlaidlaw@kingsmeadowsmedical.com.au; 3 Kings Meadows Medical Centre, Kings Meadows, TAS 7249, Australia

**Keywords:** homelessness, healthcare, nursing, nurse-led

## Abstract

People who are homeless experience significantly poorer health than the general population and often face multifaceted challenges engaging with public healthcare services. Mission Health Nurse-led Clinic (MHNC) was established in 2019 to meet the healthcare needs of this marginalised population in Launceston, Tasmania. This study examines barriers to healthcare access amongst individuals who experience homelessness, client and staff perceptions of the MHNC services and explored opportunities for service expansion. Descriptive statistics were drawn from administrative data, and all interviews were thematically analysed. A total of 426 presentations were reported for 174 individuals experiencing homelessness over 26 months. The median client age was 42 years and 60.9% were male; A total of 38.5% were homeless or lived in a supported accommodation. The predominant reasons for clinic visits included prescription requests (25.3%) and immunisations (20.1%). A total of 10 clients and 5 City Mission staff were interviewed with three themes emerging from the findings: personal vulnerability, disconnectedness and acceptability of the MHNC. The MHNC services were reported to be highly appreciated by all clients. Mental health and allied health, extra operating hours and maintaining the flexibility of walk-in appointments were suggested as expansion areas for the service and were highlighted as ways to increase engagement for improved health outcomes. Continued partnerships with interprofessional primary healthcare providers would contribute to addressing unmet healthcare needs in this vulnerable population.

## 1. Introduction

Homelessness has long been recognised as a significant global social issue associated with serious and long-term health implications [1,2,3]. Research consistently shows that homeless individuals are more commonly affected by tri-morbidity—the combination of mental illness, physical illness and drug or alcohol misuse—than those individuals who are not homeless [4,5], and they often seek care only at an advanced stage of health deterioration [2,6]. During the current COVID-19 pandemic, there has been a growing impetus for governments, both local and federal, to address the health concerns of homeless persons with more political will [7,8] and to source sustainable solutions. The homeless experience disengagement with primary healthcare services, commonly presenting to local emergency departments (EDs) for healthcare needs, due to challenges relating to access [9], inadequate transportation, and also due to encountering a variety of other barriers such as stigma and discrimination, low health literacy, competing demands and financial constraints [4,6,10,11,12]. Additionally, there is a deficit of primary healthcare providers, and very few offer bulk-billed or free services. One innovative way to overcome issues related to healthcare access for people experiencing homelessness is through the establishment of outreach nurse-led services [1]. Despite there being clear benefits of nurse-led care in reducing the burden on local emergency departments [13,14,15], there is little research exploring whether such interventions are helping to meet the health service needs of regionally based vulnerable and homeless populations. The Mission Health Nurse-led Clinic (MHNC), established in March 2019, is a nurse-led clinic that aims to address the inequitable accessibility and provision of timely healthcare for persons experiencing or at risk of experiencing homelessness in Launceston Tasmania, Australia. The MHNC is a collaborative initiative between an independent nurse practitioner, City Mission Launceston and the School of Nursing, University of Tasmania. The clinic operates one morning per week and is routinely staffed by a nurse practitioner (NP) and/or a registered nurse (RN). Healthcare services are provided within the scope of practice of the NP and RN, including health assessment, prescriptions, treatment of minor illnesses and patient education. Its co-location with an overnight emergency shelter service, the nurse-led model of care is proving effective in addressing the healthcare needs of individuals experiencing homelessness within a regional context. This study identifies the complexities of accessing equitable and timely healthcare for persons experiencing homelessness, from the perspectives of the clients themselves and from those of the staff providing the MHNC service. The uniqueness of this study, incorporating the perceptions of both the persons who utilised the healthcare service and those of the service providers, adds further dimension to the complexities of homelessness and adds to the growing body of knowledge surrounding this growing global phenomenon. Further opportunities for expanding the breadth of nurse-led healthcare services are also explored.

## 2. Materials and Methods

### 2.1. Study Design

To explore barriers in accessing healthcare amongst homeless people and benefits of the MHNC services, this study followed the principles of a mixed methods evaluation design. This method uses qualitative and quantitative analysis to evaluate programs [16]. In this study, the qualitative data was collected to determine client and staff perspectives of current and future MHNC services through face-to-face interviews from March–May 2021. Quantitative data were exported from administrative management software from March 2019 to May 2021 (26 months) and included clients’ age, gender, living situation, previous medical history, reason for presentation, number of visits to MHNC and where clients were referred to. The final stage of this study was combining findings to inform and support future recommendations to meet the needs of a socioeconomically disadvantaged population.

### 2.2. Qualitative Data Collection and Analysis

**Figure 1:**
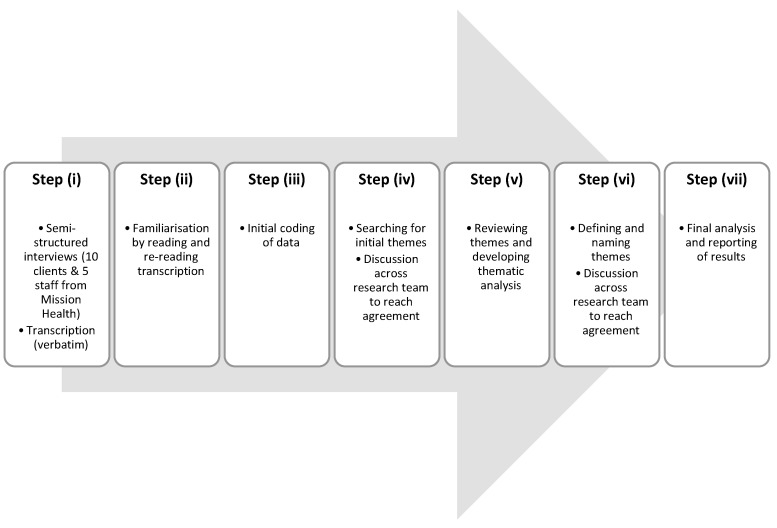
Overview of the analysis process.

Purposive convenience sampling was used to recruit six to twelve clients, and four to six Mission Health staff or those affiliated with Mission Health. Purposive sampling does not necessitate the recruitment of a large number of participants if participants are familiar with the research subject [17]. All participants in this study were familiar with MHNC and able to contribute information relevant to the clinic operation and service values. Clients were eligible to participate if they were (i) 18 years old and over, (ii) had access to at least one of the MHNC services, were (iii) voluntary participants and (iv) able to give informed consent. Any current employees of the City Mission branch where the MHNC is located, including MHNC staff, were eligible to participate if they voluntarily agreed. Face-to-face interviews with clients and staff of Mission Health were conducted and recorded by two members of the research team, (GBD and LH), who had combined experience as both RNs and university academics. These two members had been involved in delivering services in the clinic since the beginning of its operation, although their duty was ceased prior to the time of qualitative data collection, and no professional relationship existed between interviewers and any participants. Recordings were transcribed verbatim by one member of the research team (MU) and then reviewed for accuracy by two researchers (GBD and KT). Thematic analysis of the transcripts was inductive and conducted using NVIVO (release 1.2, 2020) and followed the seven stages set out by Braun and Clarke [18], as outlined in Figure 1.

To ensure the rigor and trustworthiness of this study, we used member checking, coding reliability through seeking consensus and peer debriefing [18]. The research team consisted of four members familiar with the clinic context and three others with doctoral qualifications and experience in qualitative research methods. Member checks were frequently conducted during all interviews by asking participants to verify what they said. All participants were offered a chance to return the MHNC to review their transcriptions and add or correct their responses. However, none of them did so. All participants were de-identified and assigned a unique identifier. As part of step (ii), G.BD and MU independently read and re-read each transcript. After immersing themselves in the data, G.BD and MU then generated initial codes independently, as per step (iii) and discussed any discrepancies and refined the themes until consensus was reached. This process of refinement reflected phase iv. Two members of the research team had extensive knowledge and experience in the provision of healthcare among homeless populations (MD and JL) and were not involved in the initial analysis and coding but assisted in steps v–vii (Figure 1) to further identify and define themes.

### 2.3. Quantitative Data Collection and Analysis

Quantitative data consisted of administrative data from MHNC’s management software, and client information was unidentifiable to the research team and included age, gender, living situation, previous medical history, reason for presentation, number of visits to MHNC and where clients were referred to over a 26-month period. Descriptive analysis was conducted to determine frequencies in presentations numbers, and percentages for demographic data and medical conditions were used. Medians and 95th percentiles were used to report client participant age. Analysis was conducted using Statistical Package for Social Sciences (SPSS, v27, IBM Corp, Armonk, NY, USA).

### 2.4. Ethical Considerations

Ethics approval was granted from the Tasmanian Human Research Ethics Committee (H021679), and site approval was provided by City Mission. Eligible clients were determined by an administrative staff of the MHNC. Participants were provided with an information sheet about the study, and if they voluntarily agreed to participate, they were required to sign a written informed consent prior to an individual interview. This process followed the ethical considerations for working with a vulnerable population.

## 3. Results

### 3.1. Qualitative Findings

A total of 15 participants were recruited. Ten were MHNC clients, of which six were male and four were female. A total of five MHNC employees were interviewed; of these participants, three were male and two were female.

**Figure 2:**
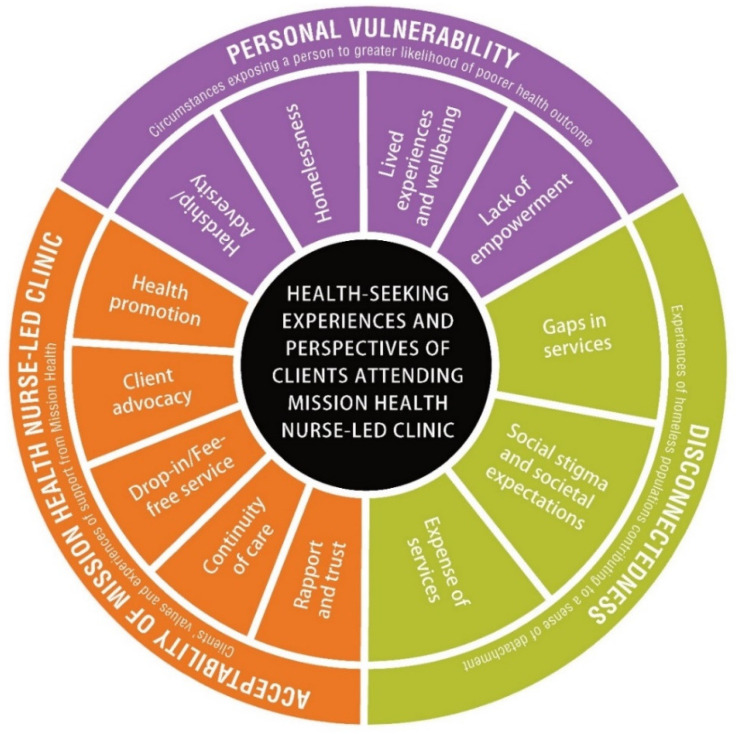
Health-seeking experiences and perspectives of clients attending Mission Health Nurse-led Clinic.

Three overarching themes developed during analysis. The first theme was personal vulnerability (client level), second, disconnectedness (system level) and third, the acceptability of the MHNC (service level). Definitions for each overarching theme and a list of subthemes are provided in Figure 2.

#### 3.1.1. Personal Vulnerability

Personal vulnerability, as an overarching theme, was identified from client narratives that told of a loss of self-esteem, powerlessness, high stress and social isolation. Hardship and adversity, homelessness, lack of empowerment and lived experiences and wellbeing were identified as subthemes.

##### Hardship and Adversity

Hardship and adversity were discussed by more than half the study participants, with various life experiences contributing to this difficulty such as limited social connection, poor mental health and/or little or no financial safety nets. Comments from clients included:

“They haven’t walked a day in my shoes, or any of our shoes so there’s no way on God’s earth that they can tell us it’s gonna be alright … they don’t have a clue till they walk in our shoes”.

(MHNC client, 1)

“I didn’t have a lot of money … I get a lot of stuff stolen off me so I usually end up with not much money and then because I couldn’t pay [for a doctor’s appointment] they have declined to meet my essential needs as a human being”.

(MHNC client, 7)

Staff also identified hardship and adversity as contributing to poorer health outcomes:

“A couple of those are [domestic violence] clients and they get very anxious and upset if they’re sitting in a waiting room full of people. So, some of them just won’t go anywhere until they can come on a Thursday to Mission Health”.

(MHNC staff, 13)

“Mental health is a big issue, alcohol and drugs are a big issue, the fact that they are homeless is a big issue on their health, the Mission Health’s focus is to provide some of those basics to those health needs to help overcome the challenges.”.

(MHNC staff, 13)

##### Homelessness

Homelessness also contributed to clients’ vulnerability; one client identified the challenges of not having access to personal documents:

“[Homeless people] don’t have any paperwork, they don’t have any ID, you don’t have any of that, they can’t get the help that they need but through this [Mission Health] they can”.

(MHNC client, 7)

A staff member identified a lack of understanding by “most” people towards those who experience homelessness. They explained a lack of understanding for

“… people that are either homeless or socially isolated or [unable to] manage their lives in a way that most people would regard as normal”.

(MHNC staff, 13)

##### Lived Experiences and Wellbeing

Clients’ lived experiences and wellbeing was the third subtheme under personal vulnerability; for clients, these ranged from childhood to recent experiences with healthcare services.

“[I’ve] just been struggling so hard with mental health and self-harm since I was a boy, I’ve got the scars to prove it”.

(MHNC client, 1)

“Oh, my mental health, I suffer from severe depression…. I end up being in bed for weeks at a time. So, sometimes I miss my appointments because I’m not in the right place to go there and as medical practitioners they should be aware of that”.

(MHNC client, 7)

“In Tasmania in general…I’m hearing stories…people are not happy, not happy about [the lack of mental health services]. Personally, I’m one of ‘em. I’ve had to wait a month to see a mental health counsellor….a lot can happen within a month”.

(MHNC client, 10)

Staff also discussed their experiences of dealing with clients of the MHNC and identified a

“… need for primary health services delivered in a setting and a situation that is acceptable to [their clients]”.

(MHNC staff, 2)

##### Lack of Empowerment

The fourth subtheme was a lack of empowerment where clients discussed feelings of anxiety and social inadequacies in their opportunity to communicate their needs to medical staff:

“You know, once you lose the loop of socialising you do find it hard to get help. … I did see a lady [GP], but she retired, she was really good and listened to what I had to say”.

(MHNC client, 1)

“Because there’s a lot of people out and about on the streets that definitely need the medical attention and help but they won’t sit in a hospital, they can’t build a rapport with a normal doctor [GP]”.

(MHNC client, 6)

One of the City Mission staff also identified a lack of empowerment experienced by the clients of Mission Health in accessing multiple healthcare services in multiple locations:

“… if they’re in a sensitive state, you know, a mental health state, then they don’t necessarily have it in them to chasing multiple locations and multiple services”.

(MHNC staff, 15)

#### 3.1.2. Disconnectedness

Disconnectedness was the most frequently discussed themes and has been defined one of the experiences of homeless populations that contributes to a sense of detachment from health services. Subthemes included gaps in or between services, social stigma and societal expectations, as well as the expense of alternative health services.

##### Gaps in Services

The first and most frequently discussed subtheme under disconnectedness was gaps in or between services. Eight of the ten clients and all staff interviewed highlighted this as a significant challenge among the study population:

“I was with [another medical service] for a long time and then my doctor passed away and my other doctor retired after a two-year rapport and then I was just going here, there and anywhere”.

(MHNC client, 6)

“It seems that it’s difficult to access general health care apart from emergency or ambulatory care in this town, in this region even”.

(MHNC client, 10)

Staff discussed how clients used the emergency department for “something that’s relatively minor” (MHNC staff, 15) more frequently before the MHNC was established and expressed frustration in the challenges they experienced when trying to support a client in seeking healthcare:

“We often used to hear of them just presenting at emergency at the hospital when they [had] certain conditions because they know when they turn up there and they wait long enough they will see someone”.

(MHNC staff, 12)

“We wouldn’t have a referral process. We’d just say [to clients], ‘… just try your luck there’ basically. We’d get reports back saying, ‘[GP’s are] not taking on new clients’”.

(MHNC staff, 13)

##### Social Stigma and Societal Expectations

Clients of MHNC also discussed experiencing social prejudice and felt the stigma attached to that along with societal expectations when seeking other forms of healthcare but indicated that they did not experience this when presenting to MHNC. When asked what barriers he had faced when seeking healthcare, one participant (MHNC client, 7) described it as “financial bigotry or financial racism”, which he described as being “judged because you’re broke”. Another client, when describing a conversation with a person he referred to MHNC, he stated

“Don’t’ be so silly, just tell ‘em [MHNC nurses] who you are, tell‘ em the story. They don’t care about your smelly feet”.

(MHNC client, 1)

Another client discussed his own experience with hospital and general practice services:

“The last time I went to the doctor I felt like cattle … I’m somewhat disillusioned”.

(MHNC client, 10)

The research team also interviewed a client whose experience of social stigma and societal expectations was described as a barrier to accessing health services:

“[With general practice services] it’s difficult for them to just attend at a certain time and sit in a waiting room and not sort of rush from the place or not turn up”.

(MHNC staff, 12)

##### Expense of Services

The third subtheme under ‘disconnectedness’ was the expense of services and was discussed by three clients and two staff of MHNC. Clients identified both the cost of attending or missing general practice appointments as prohibitive:

“Well, it costs money, cos my family doctor, she doesn’t bulk bill anymore”.

(MHNC client, 2)

“I’ll make appointments with best intentions and then don’t rock up and get a $50 (AUD) charge for it and still don’t get to see the doctor”.

(MHNC client, 6)

Staff participants were also concerned about service affordability:

“Most of our clients [don’t] have the money to pay the gap … there wasn’t really places we could send because most of the clients who come to us have no money”.

(MHNC staff, 11)

#### 3.1.3. Acceptability of Mission Health Services

Acceptability of Mission Health was the third theme identified; this was defined as the clients’ values and experiences of support from the MHNC. Five subthemes were constructed under this overarching theme: (i) rapport and trust, (ii) continuity of care, (iii) drop-in and fee-free service, (iv) client advocacy and (v) health promotion.

##### Rapport and Trust

The most frequent subtheme discussed by client participants was rapport and trust, which was discussed by four client and two staff participants.

“…here [at MHNC] they can see the same people every week. And, like I said build a rapport”.

(MHNC client, 6)

“They actually listen to me. They don’t judge you, like there’s all walks of life that come here but most of us are drug addicts, alcoholics or victims of one sort or the other”.

(MHNC client, 1)

A female client participant with long-standing mental health problems who used the MHNC service regularly stated:

“Well, they’ve [Mission Health staff] saved my life and that’s, and that’s very humbling”.

(MHNC client, 7)

Staff participants had also witnessed how rapport and trust contributed to the appropriateness of MHNC, as they stated:

“We had a gentleman come in the other day with his three-year-old grandchild left in his care and he had no idea what to do. He didn’t have the money to take her to a doctor, and they’re just so grateful. You know, it’s people’s health, if you haven’t got your health what have you got?“

(MHNC staff, 15)

##### Continuity of Care

Continuity of care for clients of MHNC was the most frequent theme discussed by staff, with four staff and three clients providing statements used to inform this subtheme. Staff identified the benefits of providing a service with regular staff who understood the complex nature of the clients they were seeing:

“A lot of our clients have multiple complex needs. A five-to-seven-minute session with your [GP cannot] possibly identify what they actually do need”.

(MHNC staff, 11)

Staff also identified a lack of continuity of care in previous health-seeking practices of clients, resulting in many opting to present to the local emergency department.

Client participants also valued the opportunity to consistently see the same NP and RNs at MHNC and valued being referred to appropriate services as needed:

“Since I’ve been in Launceston for the last six months, I’ve been coming here to see the [nurses] every Thursday when I can”.

(MHNC client, 1)

“I’m very happy with the services that I’ve got because they can refer you for x-rays, higher specialists. So no, for me they’ve covered everything I’ve possibly needed. But they’ve referred me through and given me the help and support I’ve needed to try and look after myself”.

(MHNC client, 7)

##### Drop-In/Fee-Free Service

Clients of MHNC valued the opportunity to be able to drop in and receive a fee-free service:

“I’m not reliable or good with meeting appointment times, here I drop in and have a coffee and just wait and that works for me rather than being structured to a time that doesn’t necessarily work for me”.

(MHNC client, 6)

“And if ya haven’t got much money you can just come here and get help”.

(MHNC client, 4)

One staff participant also discussed this aspect of the service design as being valued by clients:

“Within the cohort that we service I think that they really appreciate the fact that it’s gonna be free … I think they like the fact that they can just turn up without a lot of organisation, they don’t have to pre-book and that sort of thing. You know that’s an opportunity just to turn up and see if you can be treated”.

(MHNC staff, 12)

##### Client Advocacy

Client advocacy was the fourth subtheme contributing to evidence of the acceptability of MHNC. One client discussed how he advocated for others to attend the service:

“I will actually come with them on the first appointment just to get them in that door”.

(MHNC client, 7)

One staff member (MHNC staff, 14) also discussed the importance of advocacy for the clients using MHNC:

“There’s just that whole advocacy of their health management that [MHNC clients] need … the great thing I think with the nurse practitioner is that they do really advocate well [for clients]”.

(MHNC staff, 14)

##### Health Promotion

The fifth and final subtheme was health promotion, which was discussed by two staff participants. These staff members identified the opportunity to encourage clients attending other City Mission service to call into the MHNC; one staff member (MHNC staff, 14) explained what she has said to clients, “If [they’re] at the service and they’re here having a meal, we can say, ‘well, why don’t you book in for a flu vaccination?’” The participant went on to explain:

“It certainly is identifying all the health issues and we are able to treat, we’re fortunate to have a nurse practitioner [here] so we are able to do more than we could achieve without a nurse practitioner”.

(MHNC staff, 14)

#### 3.1.4. Client and Staff Recommendations

Client and staff participants were also asked to provide recommendations for the MHNC. The most frequently discussed need was the provision of mental health support through the addition of social workers, psychologists, psychiatrists and drug and alcohol support services. Increased hours for the MHNC were also recommended by staff participants. Other recommendations included regular on-site consultations by various allied health services such as dentistry, podiatry and occupational therapy, as well as the regular availability of a diabetic educator and medical practitioner. Staff participants also suggested a holistic approach across the lifespan in healthcare service provision for this vulnerable population. The need for continued support from key stakeholders was also identified to ensure service sustainability. One participant concluded their interview by saying:

“It all just [needs to] happen in the one place. I think for a lot of people, the continuity and stability and availability is really important”.

(MHNC staff, 15)

### 3.2. Quantitative Results

Over a period of 26 months, 174 clients presented to the MHNC 426 times. A summary of age, living situation, medical history and the reason for presenting are provided in Table 1. Briefly, the median client age was 42 years; 60.9% were male; and 38.5% were homeless or lived in a supported accommodation. A significantly higher proportion of males fitted our definition for homelessness (*n* = 106, 47.2%) versus females (*n* = 68, 23.5%), and this difference was significant at *p* = 0.002. Mental health issues and substance/alcohol abuse were most commonly reported in health histories (24.8% and 13.8%, respectively). The main reasons for clinic visits included prescription requests (25.3%) and immunisations (20.1%). The majority of clients (82.2%) did not require further referral, while 11.5% were referred to either radiology or pathology services. No clients were referred to the emergency department.

## 4. Discussion

This study investigated the barriers to healthcare access among people experiencing homelessness and vulnerability. The perspectives and experiences of the clients and staff of the MHNC service were investigated with regard to health among the homeless population within the context of regional Tasmania and the multiple barriers that prevent them from accessing primary healthcare in this regional location. The study identified three overarching themes: personal vulnerability (client level), disconnectedness (system level) and acceptability of the MHNC (service level). While the first two themes reflected and reinforced the well-documented literature relating to socioeconomic disadvantages and detachment from health services amongst people experiencing homelessness, this current study provides a different and holistic perspective by exploring the perception of homeless people’s vulnerability across these three levels of healthcare provision.

This study identified the following as contributing factors to a greater likelihood that a person would experience poorer health outcomes: personal vulnerability; which correlated with hardship and adversity; perceived or actual disempowerment; and traumatic lived experiences. The material deprivation that accompanies homelessness intensifies the sense of stigma and the loss of self-esteem and hope, which engenders personal vulnerability [19]. Various life experiences contribute to this difficulty such as limited social connection, poor mental health, high rates of substance abuse/misuse and/or little or no financial safety nets. Other studies also support these findings [20,21,22] and emphasise the challenges to meet healthcare needs amongst people experiencing or at risk of homelessness.

Disconnectedness from health systems was a pronounced finding, with the participants expressing that their use of mainstream health services was fragmented and haphazard. The reasons for their detachment were attributed to not only financial constraints but also that they felt societal stigmatisation precluded them from seeking healthcare services. Gaps in or between services were highlighted by eight of the ten clients and interestingly, by all staff. Disconnectedness or detachment from mainstream health services has been regularly identified within the reviewed literature [15,20,23].

The experiences of social stigma, societal expectations and the expense of healthcare services have also been reported by Nyamathi, Salem [14] and Easterday, Driscoll [24]. This socio-political and financial dimension of the experience of homelessness, although not unique to this study, was clearly articulated and defined by one of the participants (MHNC client, 7). He brings these aspects together in the discussion of his experiences with disempowerment due to reduced financial resources; he emphasised the powerful impact of this by referring to it as “*financial bigotry or financial racism”*. The individual defined this as being *“judged because you’re broke”* (MHNC client, 7).

This study brings into sharp focus the significant and unique needs of people experiencing homelessness in a regional context. The subthemes identified in this study were rapport and trust; continuity of care; drop-in and fee-free service; client advocacy; and health promotion. These are in alignment with Levesque’s definition of access and the provision of the right service, in the right place, at the right time [25].

Nurse-led clinics have a proven track record of facilitating improved access to healthcare, enhanced care continuity and the containment of associated costs [20], consequently providing effective and efficient solutions to the challenges identified in this study. In regional and rural areas RNs and NPs are perfectly placed to address insufficient healthcare resources [26,27] and to augment access to appropriate healthcare for people experiencing homelessness [20]. The discourses within the literature regarding the provision of nurse-led care for people experiencing vulnerability has been accepted by clients [26,27]. The emerging evidence within the Australian context demonstrates that healthcare provided by nurses is safe, cost effective [28,29,30], sustainable and potentially replicable in the areas of greatest need. A study by Roche et al. [20], supports our findings of the importance of appropriateness and the acceptability of nurse-led clinics in the provision of care for those experiencing homelessness.

One of the strengths of this study was the unique way of incorporating the perceptions of both the persons who utilised the healthcare service and those of the service providers, effectively providing additional information on the dimensions and complexity of homelessness. For the participants of this study, nurse-led services offered an alternative to the more traditional healthcare model, addressing the clients’ complex needs while building trust and rapport [4]. This was facilitated by the ability to provide longer consultation times than those given in general practice models [20]. A primary goal of the MHNC was to improve accessibility, availability and affordability in the provision of healthcare. The MHNC also sought to provide a sense of connectedness between those experiencing homelessness and traditional healthcare facilities. Participants offered positive experiences in the approachability of the staff and the care provided. The clients valued the acceptance and non-judgmental manner of the staff. Roche, Duffield [20] also noted in their research that clients found nurse-led clinics for persons experiencing homelessness helped to diminish the perceived social stigmatisation of this marginalised cohort through its professional and approachable atmosphere. Enhancing the sense of connectedness, the staff at the MHNC were able to address the healthcare needs of the presenting clients and effectively link them to resources for both immediate care and preventative future care.

The limitations of this study include how quantitative data were collected; it is possible that additional findings might have been reported if the administration staff had recorded data for each appointment rather than aggregating the data of all patients. Future work may benefit from a more detailed analysis of individual appointments to fully determine the healthcare needs of clients experiencing homelessness. Thematic analysis was used to analyse interviews, and the research team was focused on rigorous evaluation with review at various stages across the research team to ensure the consistency and cohesion of qualitative findings.

This study looked at objective measures to determine the effectiveness of the MHNC to address the complexities of adverse health outcomes for vulnerable persons and those experiencing homelessness. This study offers in-depth comprehension of the health challenges faced by the homeless cohort provides data and insight into where supports may be best provided to enhance both access and engagement with services and warrant innovative and sustainable solutions. These insights can be applied to inform not only future healthcare clinics for persons experiencing homelessness but can also be used to inform health policy, educational programs and the general populace on the health provision needs of this marginalised group. Furthermore, while the study was useful in demonstrating that a specific nurse-led service was well received and offered a quality alternate healthcare pathway to the more traditional provisions of care, further research to inform the sustainability of the MHNC and in particular, a critical economic evaluation, are recommended.

## 5. Conclusions

For persons experiencing homelessness the barriers of seeking healthcare can be incredibly complex. This study has demonstrated that a collaborative model of nurse-led healthcare can mitigate the challenges of disconnectedness with other primary healthcare services, such as improved access and equity. Additionally, while there is scope for further innovative strategies to support and improve the health of this marginalised population, this study has offered the participants a portal to be heard, demonstrating the value of the nurse-led clinic on multiple levels.

## Figures and Tables

**Table 1:** Summary of client presentations to Mission Health Nurse-led Clinic.

	No.	Median		Range (95th %)	
Total number of clients	174				
Total number of presentations	426	1		1–21 (1–9.7)	
Age		42 years		1–77 years (3.3–69.4)	
		**No.**		**%**	
Gender					
Male		106		60.9	
Female		68		39.1	
Living situation					
Home		107		61.5	
Supported		19		10.9	
Homeless		48		27.6	
**Medical condition**		**History**		**Presentation**	
		**n = 174**	**%**	**n = 174**	**%**
Medication prescription		n/a	n/a	44	25.3
Immunisation		n/a	n/a	35	20.1
Musculoskeletal (chronic or acute)		15	8.6	20	11.5
Mental Health		43	24.8	17	9.8
Skin		5	2.9	17	9.8
Respiratory conditions		15	8.6	13	7.5
Gastrointestinal/renal/urinary conditions		17	9.8	11	6.3
Other		21	12.1	10	5.8
Cardiovascular conditions		13	7.5	7	4.0
Eyes/ears/nose/throat		2	1.2	6	3.5
Substance and alcohol use		24	13.8	4	2.3
Reproductive/sexual health		n/a	n/a	3	1.7
Diabetes Mellitus		13	7.5	2	1.2
Missing/unknown		60	34.5	1	0.6
Neurological conditions		7	4.0	0	0

## Data Availability

This data are not publicly available, and ethics approval has not been provided to share data at time of publication.
